# Editorial: Graph representation learning in biological network

**DOI:** 10.3389/fbinf.2023.1222711

**Published:** 2023-06-09

**Authors:** Swarup Roy, Pietro Hiram Guzzi, Jugal Kalita

**Affiliations:** ^1^ Network Reconstruction & Analysis (NETRA) Lab, Department of Computer Applications, Sikkim University, Gangtok, India; ^2^ Department of Surgical and Medical Sciences, Data Analytics Research Centre, Magna Graecia University, Catanzaro, Italy; ^3^ Department of Science, University of Colorado, Colorado Springs, CO, United States

**Keywords:** graph, representation learning, embedding, complex network, regulatory network, protein network

Network modeling is a powerful alternative in computational biology for deciphering valuable and novel insights into complex molecular interactions and functions of biological systems (Guzzi and Roy, 2020). Network Science is a new paradigm of understanding complex systems through graph formalism. Network biology is used in pharmacology and drug discovery to enhance comprehension of intricate connections among drugs, targets, and diseases. This utilization facilitates the development of novel molecules or the identification of repurposed drugs. Representation learning (RL) has recently found its niche for graph-structured data with state-of-the-art results for various domains. With the record of successes in network biology, there has been a tremendous surge in leveraging representation learning for biological networks, from modeling to learning with networks. At its core, the spectrum of algorithmic approaches facilitated with a trained behavior over topological features converts networks into vector spaces. Leveraging machine and deep learning, in particular, for graph learning has revolutionized the task of graph representation across different domains.

The number of applications produced by these vector spaces is paramount as they yield in performing tasks like predicting missing links in a graph, graph clustering, classification, generation, alignment, *etc.* Inherently, the notion of representation learning for biological networks reflects an optimized view of network topology over the algorithmic paradigm of hand-curated and hard-coding feature techniques that lack to understand the higher-order structures and often fail to embrace the inductive capability as they do not influence network information into the predictive models. Therefore, the proposition of representation learning models for biological networks of complex design and the multimodal topological structure emerges as a suitable direction for future advancements. This research proposes to find efficient representations for biological networks and solve network-related learning tasks. RL is a potential game changer in more precisely deciphering the inherent complex interaction patterns in a complex network.

Despite the recent introduction of such methods, the increasing interest of the scientific community has consolidated these approaches. Consequently, there are many applications of graph representation learning to model and analyze massive and multimodal biological data generated from high-throughput omics technology, epidemiological data, and electronic health records. Integrating multiple data sources is a booming research area in the graph representation learning domain. Such approaches usually integrate many data sources in the same graph and then try to learn the best representation to uncover hidden knowledge from data. In the medical domain, this may be used to diagnose rare diseases. This Research Topic highlights novel research in network biology coupled with representation learning and its application in biology, medicine, and pharmacology.

Da Silva Lopes et al. presented the application of graph representation learning in a disease study (Ferreira et al.). Authors apply graph-based classifiers to diagnose and treat a rare disease starting from a graph obtained from the analysis of protein structures. There are many approaches to analyzing protein structures through graphs, such as protein contact networks and protein residue networks. Such models represent single elements of the proteins (amino acids, substructures, and atoms of the backbone) as graph nodes, while the edge encodes the spatial distance among elements. Similarly, the transformer network model may be applied to study quantitative structure–activity relations (QSARs), as presented by Wang et al. The authors provide a graph neural network (GNN) and a graph subgraph transformer network (GSTN) to study the screening of cardiotoxic compounds. The authors use a transformer network to model both the structure and activity of compounds, represented as graphs. Then, the transformer is used to mine such data and classify compounds based on cardiotoxicity. The existence of many approaches requires the introduction of systematic reviews to provide both researchers and practitioners a valuable bag of tools to mine data; the mini-review of Petrizzelli et al. presents a bag of tools available for the analysis of protein dynamics to help practitioners and researchers in this field.

These approaches share the common idea of encoding the network structure, represented as the graph’s adjacency matrix, into a latent low-dimensional space. Such methods are based on many algorithms, from matrix factorization to deep learning and complex non-linear models based on non-Euclidean geometries. The relevance of these methods is the possibility to efficiently map in a low-dimensional space, both topology and biology (i.e., all the available metadata for nodes and edges). In this case, the same latent space may represent genes, proteins, and ontological concepts. The article by Gervits and Sharan presents the application of variational autoencoders to predict genetic interactions, cell line dependencies, and drug sensitivities. Variational autoencoders (VAEs) are a class of probabilistic generative models composed of two neural networks called the encoder and decoders. The first network map input data into a latent space, while the second can map encoded data into the original space. Autoencoders can predict missing links in graphs. In this article, the authors use the VAEs to recover missing links between genes, cell lines, and drug information.

We are delighted to announce the publication of a Research Topic on “*Graph representation learning in biological network*” in Frontiers in Bioinformatics. This Research Topic aims to showcase the latest research and advancements in graph representation learning and its applications in biological networks. The Research Topic contains high-quality manuscripts covering many different applications. The aforementioned high-quality manuscripts will significantly enrich the knowledge base of researchers already working in similar domains or the nascent research stage in graph representation learning.
